# *Phytophthora capsici* sterol reductase PcDHCR7 has a role in mycelium development and pathogenicity

**DOI:** 10.1098/rsob.210282

**Published:** 2022-04-06

**Authors:** Weizhen Wang, Fan Zhang, Sicong Zhang, Zhaolin Xue, Linfang Xie, Francine Govers, Xili Liu

**Affiliations:** ^1^ Department of Plant Pathology, College of Plant Protection, China Agricultural University, Beijing, People's Republic of China; ^2^ Laboratory of Phytopathology, Wageningen University & Research, Wageningen, The Netherlands; ^3^ State Key Laboratory of Crop Stress Biology for Arid Areas, College of Plant Protection, Northwest A&F University, Yangling, People's Republic of China

**Keywords:** oomycete, *Phytophthora*, DHCR7, sterol, reductase, mycelium development

## Abstract

The *de novo* biosynthesis of sterols is critical for the majority of eukaryotes; however, some organisms lack this pathway, including most oomycetes. *Phytophthora* spp. are sterol auxotrophic but, remarkably, have retained a few genes encoding enzymes in the sterol biosynthesis pathway. Here, we show that *PcDHCR7,* a gene in *Phytophthora capsici* predicted to encode Δ7-sterol reductase, displays multiple functions. When expressed in *Saccharomyces cerevisiae*, PcDHCR7 showed the Δ7-sterol reductase activity. Knocking out *PcDHCR7* in *P. capsici* resulted in loss of the capacity to transform ergosterol into brassicasterol, which means PcDHCR7 has the Δ7-sterol reductase activity in *P. capsici* itself*.* This enables *P. capsici* to transform sterols recruited from the environment for better use. The biological characteristics of *ΔPcDHCR7* transformants were compared with those of the wild-type strain and a *PcDHCR7* complemented transformant, and the results showed that *PcDHCR7* plays a key role in mycelium development and pathogenicity of zoospores. Further analysis of the transcriptome indicated that the expression of many genes changed in the *ΔPcDHCR7* transformant, which involve in different biological processes. It is possible that *P. capsici* compensates for the defects caused by the loss of *PcDHCR7* by remodelling its transcriptome.

## Graphical abstract

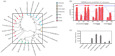

## 1. Introduction

Sterols are a class of important lipids in most eukaryotes and some prokaryotes, where they may play a key role in maintaining the integrity and fluidity of cell membranes, as well as regulating biological processes [1]. Sterol biosynthesis and sterol composition have been well studied in a number of organisms, and the overall picture that emerged is that the multistep biosynthesis process relies on a series of rather conserved enzymes, next to enzymes that are specific for certain lineages. As a result, the end products are different, with variations in side chains and double bonds of multiple rings. Within eukaryotes, fungi have ergosterol as the main sterol, vertebrates produce cholesterol, *Dictyostelium* species have dictyosterol, and in plants stigmasterol, campesterol, β-sitosterol and brassicasterol are the most common sterols [2]. In a few prokaryotes, sterols such as lanosterol and cycloartenol were identified [3,4]. For most eukaryotes, sterols are vital for their survival, and it is therefore not surprising that compounds classified as sterol biosynthesis inhibitors (SBIs) have a large share in the fungicide market, especially in agricultural production for the management of fungal plant pathogens [5].

Despite the vital role of sterols, some organisms are sterol auxotrophic; they cannot synthesize sterols and have to recruit exogenous sterols via ingestion or absorption. Sterol auxotrophs include nematodes, most arthropods, many ciliates and some genera of oomycetes [6–9]. Oomycetes are a diverse group of eukaryotic micro-organisms in the Stramenopile lineage that comprises quite a number of devastating filamentous plant and animal pathogens [10,11]. Well-known oomycetes are *Phytophthora* species, important plant pathogens that cause serious damage in agriculture, forests and natural ecosystems. Examples are *Phytophthora infestans,* the causal agent of late blight and also known as the Irish potato famine pathogen [12], *Phytophthora ramorum*, causing sudden oak death and ravaging forests in the USA and the UK [13], and *Phytophthora capsici,* a species with a wide host range and extremely devastating in many vegetable crops [14,15]. Although traditionally classified in the kingdom Fungi, oomycetes are phylogenetically distinct from true fungi. They have evolved independently and this is also reflected at the biochemical and physiological level [16]. Fungi, for example, are sensitive to SBIs; they produce large amounts of ergosterol which is essential for the plasma membrane integrity. By contrast, *Phytophthora* species are not sensitive to SBIs because they are sterol auxotrophic, and the same holds for species in the oomycete genus *Pythium*, also mainly plant pathogens, and the obligate downy mildew pathogens. For a long time, all oomycetes were thought to be sterol auxotrophic, but in the last decades, this is called into question. Studies inspired by sterol profile analyses and genome sequencing demonstrated that genera comprising largely animal pathogens like *Aphanomyces* and *Saprolegnia* do possess the capacity to synthesize sterols, and as in fungi, some SBIs can strongly inhibit their growth [17,18]. It is thus conceivable that the last eukaryotic common ancestor (LECA) of oomycetes had the capacity to synthesize sterols.

Even though *Phytophthora* is sterol auxotrophic, at least two genes encoding enzymes in the sterol biosynthesis pathway were identified in different *Phytophthora* species, i.e. *ERG3* and *DHCR7* [2,19]. The enzyme ERG3 is a C-5 sterol desaturase while DHCR7 is a Δ7-sterol reductase. In *de novo* sterol biosynthesis in eukaryotes, Δ7-sterol reductase can remove the double bond at the seventh carbon of Δ7-sterols. In animals, DHCR7 is responsible for converting 7-dehydrocholesterol into cholesterol, which is the final step of cholesterol synthesis in the Kandutsch–Russell pathway [20]. In humans, mutations in DHCR7 may lead to the Smith–Lemli–Opitz syndrome, a common recessive genetic disorder, causing among others, developmental defects and mental retardation [20,21]. In plants, the homologue of DHCR7 named DWARF5 is crucial for brassinosteroid biosynthesis and, as the name implies, the loss of function results in dwarfism of *Arabidopsis* [22]. As yet, the function of DHCR7 in sterol auxotrophic organisms has not been studied.

The aim of this study was to investigate the function of DHCR7 in a sterol auxotrophic *Phytophthora* species. We chose *P. capsici* as a model species because of its importance as plant pathogen and because it is amenable to gene editing using CRISPR/Cas9 [23]. *P. capsici* causes root, crown or fruit rot in over 20 families of plants including Cucurbitaceae, Solanaceae and Leguminosae [15,24]. Its life cycles can be divided into sexual and asexual ones. In the sexual life cycle, *P. capsici* can produce long-lived dormant oospores, which can be used as the main source of preliminary infection in soil. *P. capsici* is heterothallic and thus needs two strains with different mating types for sexual reproduction. By contrast, in the asexual lifecycle, the branched sporangiophores emerged from hyphae can produce sporangia, and the mature sporangia can quickly release biflagellate motile zoospores that swim chemotactically and infect plants, which are the main resources for secondary infection and the spread of this disease [15]. In this study, we characterized the *P. capsici DHCR7* gene (*PcDHCR7*), analysed its expression during the *P. capsici* life cycle and verified the enzyme activity of DHCR7 by heterologous expression in yeast. Moreover, we analysed the phenotypes and the sterol-modifying capacity of *PcDHCR7* knock-out transformants and revealed molecular and biological functions of this gene in *P. capsici*.

## 2. Results

### 2.1. Characterization of DHCR7 and its expression profile in *Phytophthora capsici*

**Figure 1.**
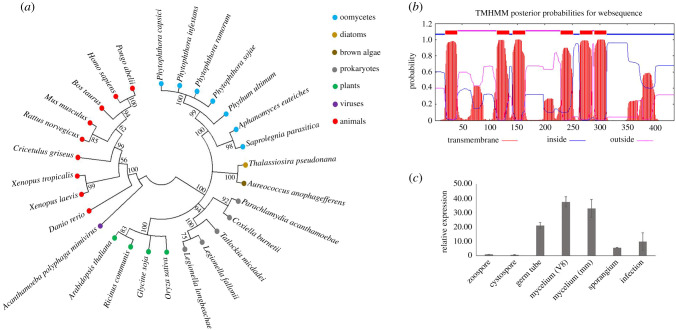
Characterization of DHCR7 protein and its expression profile in *Phytophthora capsici*. (*a*) Condensed molecular phylogenetic tree of DHCR7 protein sequences of representative species from different lineages. Bootstrap values are expressed as percentages based on 1000 repetitions, and only those with greater than 50% branch support are shown. (*b*) Transmembrane domain analysis of PcDHCR7 using TMHMM. Six transmembrane domains are predicted to be present in the PcDHCR7 protein. (*c*) Expression profile of *PcDHCR7* in *P. capsici* by RT-qPCR analysis. The gene *PcDHCR7* is expressed in all development stages and during infection (4 dpi). V8 means that mycelia were cultured on V8 medium, and mm indicates that mycelia were cultured on a minimal medium without any sterol. Data represent the mean ± s.d. from three biological repeats.

Although *Phytophthora* spp. are known as sterol auxotrophs, the gene *DHCR7* was found to be present in different species in this genus [2,19]. Also in several *Pythium* species, which cannot synthesize sterols either, genome mining revealed the presence of a *DHCR7* homolog. Sequence alignment (electronic supplementary material, figure S1) and phylogenetic analysis (figure 1*a*) showed that DHCR7 is highly conserved within oomycetes and suggested that the *DHCR7* gene was present in the LECA of oomycetes and before speciation. Protein signature analysis of PcDHCR7 from *P. capsici* identified six transmembrane domains (figure 1*b*), indicating it is probably a transmembrane protein.

The fact that *P. capsici* cannot synthesize sterols raised the question if *PcDHCR7* is expressed and if so in which life stages and under which conditions. RNA was isolated from different life stages and from mycelium grown on a minimal medium without sterol and V8 medium that is made from vegetable juice containing natural plant sterols. Real-time (RT)-qPCR analyses showed that *PcDHCR7* was expressed in all tested life stages, also during infection (4 days after inoculation), but at different levels (figure 1*c*). The *PcDHCR7* mRNA levels in germ tube and mycelium were relatively high compared to the other life stages, and there was no significant difference in expression in mycelium cultured on medium with or without sterol (figure 1*c*). This shows that the expression of *PcDHCR7* is not affected by exogenous sterols and implies that PcDHCR7 may play a role in the development of *P. capsici*.

### 2.2. PcDHCR7 shows sterol reductase activity in *Saccharomyces cerevisiae*

**Figure 2.**
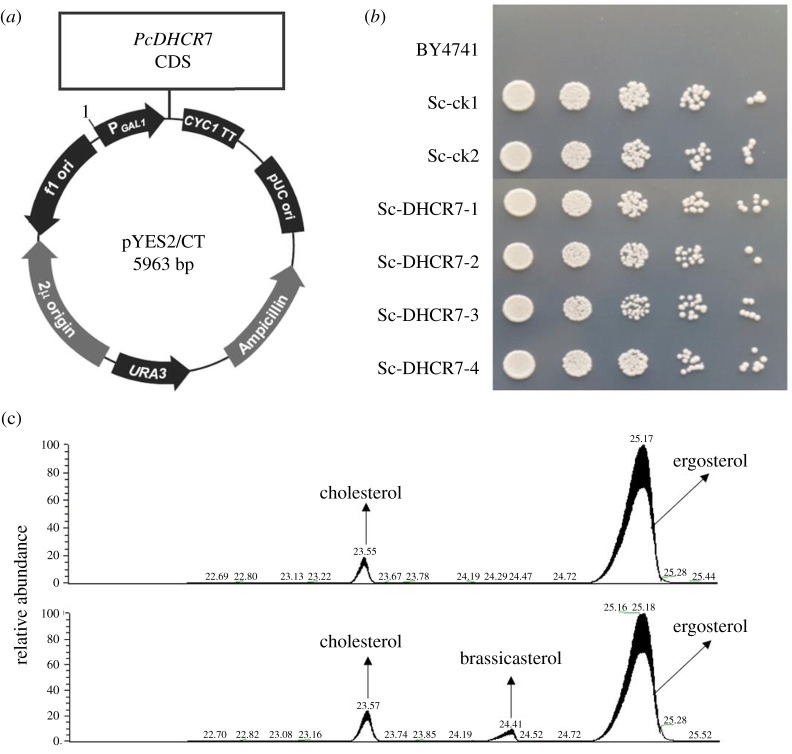
Expressing *PcDHCR7* in *Saccharomyces cerevisiae* and sterol detection from transformants. (*a*) The CDS of *PcDHCR7* was inserted into the pYES2/CT yeast expression vector under the drive of the promoter GAL1, and the original vector without *PcDHCR7* was used as a negative control. (*b*) The growth of *S. cerevisiae* transformants and parent strain BY4741 on a selective medium (which is uracil deficient) indicates that the selective marker was expressed in transformants. Empty vector strains (Sc-ck1 and Sc-ck2) had a similar growth rate to *PcDHCR7* expressing strains (Sc-DHCR7-1, Sc-DHCR7-2, Sc-DHCR7-3 and Sc-DHCR7-4). (*c*) Sterols detected from the representative empty vector strain (above) and *PcDHCR7*-expressing strain (bottom) showed that brassicasterol was present in the latter strain. Cholesterol was used as an internal standard and was manually added during sterol extraction. All the sterols indicated in the figure were detected as sterol derivatives with a trimethylsilyl at the C-3 hydroxyl.

To determine whether the protein encoded by *PcDHCR7* has a sterol reductase activity, we expressed the gene in the yeast *S. cerevisiae.* In this model organism, the sterol biosynthesis pathway is well known [25]. It lacks the DHCR7 homologue, and therefore the sterol end product—ergosterol—as well as its precursors contain a double bond at the seventh carbon. Since PcDHCR7 is a putative Δ7-sterol reductase, it might use ergosterol and its precursors as substrates. To test this, we cloned *PcDHCR7* in a yeast expression vector for heterologous expression in *S. cerevisiae* (figure 2*a*) and analysed the sterol composition of the transformants. The *S. cerevisiae* transformants expressing *PcDHCR7* showed a similar growth morphology and growth rate to those of control strains transformed with the empty vector (figure 2*b*). Hence, the presence of PcDHCR7 protein does not distinctly impair yeast growth. After 2 days' cultivation of the *S. cerevisiae* strains containing the *PcDHCR7* expressing vector as well as control strains in the liquid culture, the yeast cells were collected for sterol extraction and detection. As a result, a substantial amount of ergosterol was detected in all strains tested (figure 2*c*), while brassicasterol was found in all the strains with the *PcDHCR7* expressing vector but not in control strains (figure 2*c*). Brassicasterol is one of the important sterols in plants under natural circumstances, and the only difference between the structures of brassicasterol and ergosterol is the double bond at the seventh carbon, indicating that PcDHCR7 shows the Δ7-sterol reductase activity in *S. cerevisiae*.

### 2.3. PcDHCR7 shows sterol reductase activity in *Phytophthora capsici*

**Figure 3.**
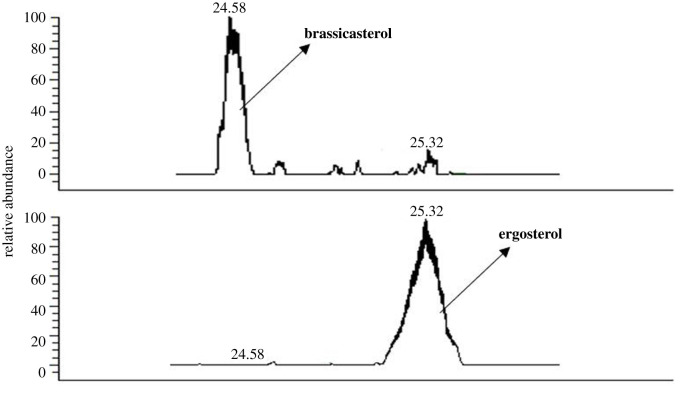
Sterol detection from different *Phytophthora capsici* strains. The wild-type strain BYA5 (above) and *ΔPcDHCR7* transformant KD1-1 (bottom) are, respectively, cultured on a minimal medium modified with 20 µg ml^−1^ ergosterol. All the sterols indicated in the figure were detected as sterol derivatives with a trimethylsilyl at the C-3 hydroxyl.

To address the question if the endogenous *PcDHCR7* gene in *P. capsici* encodes a sterol reductase that is functional in its natural setting, we chose a loss-of-function approach taking advantage of the possibilities offered by CRISPR/Cas9 genome editing [26] and obtained several homozygous knock-out transformants (electronic supplementary material, figure S2). To check the sterol reductase activity, we cultured wild-type *P. capsici* and one representative *ΔPcDHCR7* transformant on minimal medium supplemented with ergosterol at a concentration of 20 µg ml^−1^ and subsequently extracted the sterols from the mycelium. The chromatograms for sterol detection showed major differences in sterol content between the wild-type strain and the *ΔPcDHCR7* transformant*.* Brassicasterol is the major peak in the wild-type strain with almost no peak of ergosterol, whereas ergosterol is the only peak in the *ΔPcDHCR7* transformant (figure 3). These results indicate that *P. capsici* wild-type strain is capable of recruiting ergosterol from the medium and can convert it into brassicasterol. Also the *ΔPcDHCR7* transformant seems to recruit ergosterol from the medium, but in contrast to wild-type *P. capisici,* the transformant is not capable of converting ergosterol to brassicasterol. The only genetic difference between the wild-type strain and the *ΔPcDHCR7* transformant is the absence of *PcDHCR7* and hence we can conclude that *PcDHCR7* is a functional gene encoding an enzyme with the Δ7-sterol reductase activity in *P. capsici* itself.

### 2.4. Sterols saturated by PcDHCR7 promote zoospore production

**Figure 4.**
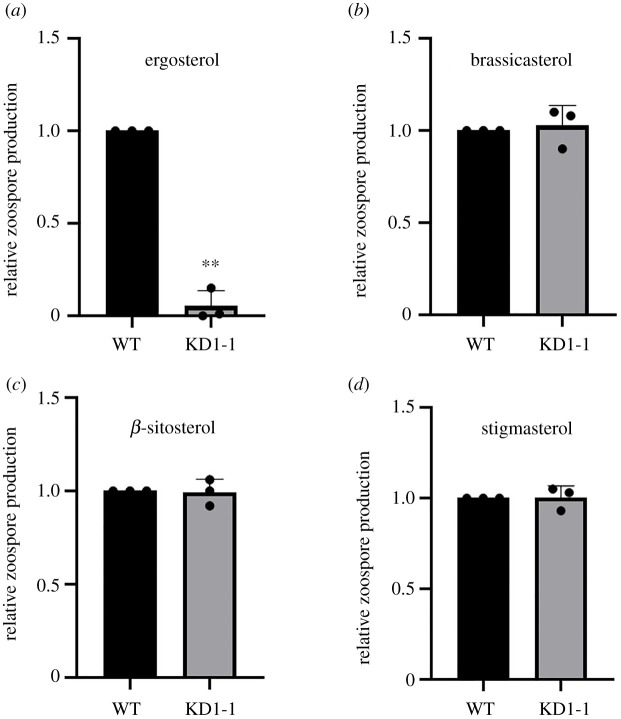
Zoospore production of *Phytophthora capsici* treated with different sterols. *P. capsici* wild-type strain and the *ΔPcDHCR7* transformant were treated with ergosterol (*a*), brassicasterol (*b*), *β*-sitosterol (*c*) or stigmasterol (*d*) with a final concentration of 20 µg ml^−1^. WT indicates the wild-type strain BYA5, and KD1-1 is a representative *ΔPcDHCR7* transformant. Zoospore production is shown as the number of zoospores of transformant relative to that of the wild-type strain. Values represent mean with s.d. of three independent experiments, and double asterisks denote significant difference from each other. **p < 0.01.

Earlier studies on several *Phytophthora* spp. have shown that adding sterols to the medium has a positive effect on growth and development [27,28]. Apparently, these sterol-auxotroph organisms can recruit exogenous sterols from the environment and use these for their own benefit. Here we tested the effect of sterols on asexual reproduction in *P. capsici.* On minimal medium without any sterol, *P. capsici* did not produce any zoospores, but when adding sterols zoospore production was triggered (electronic supplementary material, figure S3). All four tested sterols (i.e. ergosterol, cholesterol, β-sitosterol and stigmasterol) promoted zoospore production in a concentration-dependent manner while reaching a plateau at 20 µg ml^−1^ (electronic supplementary material, figure S3). With the exception of ergosterol, these sterols are downstream products of the biosynthesis step mediated by the Δ7-sterol reductase. Knowing that PcDHCR7 uses ergosterol as substrate to produce brassicasterol (as shown in figure 3) enabled us to explore whether *P. capsici* has a preference for certain sterols. When feeding ergosterol to *P. capsici*, the *ΔPcDHCR7* transformant produced hardly any zoospores while the wild-type strain showed abundant zoospore production when supplied with the same amount of ergosterol (figure 4*a*). However, when replacing ergosterol by brassicasterol, both the wild-type and *ΔPcDHCR7* transformant produced equal amounts of zoospores (figure 4*b*). These results suggest that *P. capsici* has a preference for using sterols that are saturated at the seventh carbon and this was confirmed by feeding *P. capsici* with β-sitosterol and stigmasterol which, similar to brassicasterol, are saturated at the seventh carbon. Both these sterols promoted zoospore production in the wild-type strain and the *ΔPcDHCR7* transformant at the same level (figure 4*c,d*).

### 2.5. PcDHCR7 has a role in pathogenicity of zoospores

To further investigate the role of PcDHCR7 in growth and development of *P. capsici*, we analysed mycelial growth, sporangium production, zoospore production and cystospore germination of three independent *ΔPcDHCR7* transformants (electronic supplementary material, figure S2). Compared to the wild-type strain, all three *ΔPcDHCR7* transformants showed reduced growth with significantly smaller colonies 4 days after inoculation on V8 agar medium (table 1). Other phenotypes though were not severely affected. Sporangium production, zoospore release and cyst germination rates were in the same range in the knock-out transformants and the wild-type with the exception of KD3-1 that showed a drastic reduction in sporangium production and as a consequence, also in the amount of zoospores that were released (table 1). Based on the molecular characterization, KD3-1 is a bona fide knock-out strain so why it behaves differently is not clear.

**Table 1.** Biological characteristics of the wild-type strain BYA5 and *ΔPcDHCR7* transformants.

strain	colony diameter^a,b^ (mm)	sporangium production^a,b^	zoospore release^a,b^ (×10^4^)	germination rate^b^ (%)
BYA5	68.36 ± 2.05	174.78 ± 16.93	71.67 ± 8.19	90.55 ± 1.35
KD1-1	60.08 ± 1.02**	177.22 ± 15.26	74.56 ± 9.02	90.11 ± 1.26
KD2-1	53.58 ± 2.16**	164.66 ± 13.32	70.11 ± 6.84	91.89 ± 1.39
KD3-1	50.94 ± 1.76**	55.11 ± 17.62**	17.33 ± 2.34**	90.33 ± 0.34

^a^On V8 medium.

^b^Values represent mean ± s.d. of three independent experiments, and double asterisks denote significant difference from the wild-type strain BYA5.

**p < 0.01.

**Figure 5.**
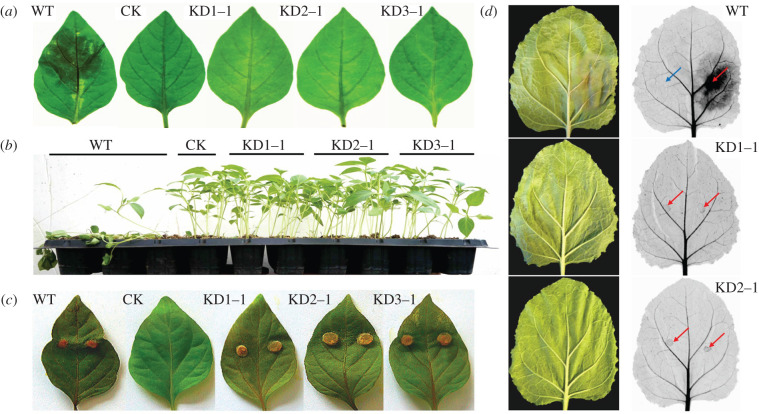
Pathogenicity evaluation of the wild-type strain and *ΔPcDHCR7* transformants with pepper and *Nicotiana benthamiana*. (*a*) Symptoms of detached pepper leaves inoculated with zoospores of different strains (3 dpi). (*b*) Symptoms of seedling pepper plants inoculated with zoospores of different strains (5 dpi). (*c*) Symptoms of detached pepper leaves inoculated with mycelia of different strains (4 dpi). WT indicates the wild-type strain BYA5; KD1-1, KD2-1 and KD3-1 are representative *ΔPcDHCR7* transformants; CK means the leaves were treated with an equivalent amount of water, or without treatment in the case of mycelium inoculation. (*d*) Symptoms of detached *N. benthamiana* leaves inoculated with zoospores of different strains (3 dpi). Blue arrow indicates inoculation site with water, and red arrow indicates inoculation site with zoospores.

Zoospore inoculation on pepper and *Nicotiana benthamiana* resulted in lesions within 3 days on leaves exposed to zoospores from the wild-type strain; however, no lesions were visible on leaves inoculated with the knock-out strains (figure 5*a*,*d*). A similar result was obtained when exposing pepper seedlings to zoospores. Five days after zoospores of the wild-type strain were added to the potting soil the seedlings wilted, but adding zoospores of the knock-out strains did not affect the health status of the plants (figure 5*b*). Using a modified CRISPR/Cas9 system, the gene *PcDHCR7* was *in situ* complemented in the *ΔPcDHCR7* transformant KD1-1 [29]. After the gene was reintroduced, the pathogenicity of zoospores was rescued partly, possibly because the expression level of the *PcDHCR7* gene in the complemented transformant was lower than that of the wild-type strain (electronic supplementary material, figure S4). Given that the cystospores of the transformants could germinate normally compared to those of the wild-type strain, it was inferred that the lack of zoospore pathogenicity of *ΔPcDHCR7* transformants was either due to aberrant invasion properties of the germ tube or to a defect in the transition from germ tube to expanding mycelial growth. Examination of inoculated *N. benthamiana* leaves using red light imaging for visualizing cell death showed that the germ tubes of *ΔPcDHCR7* transformants could still invade, but the infection was restricted to the inoculation site (figure 5*d*). This suggests that the development of *P. capsici* from germ tube into mycelium is hampered due to the lack of *PcDHCR7*.

### 2.6. Lack of PcDHCR7 affects normal mycelium development

**Figure 6.**
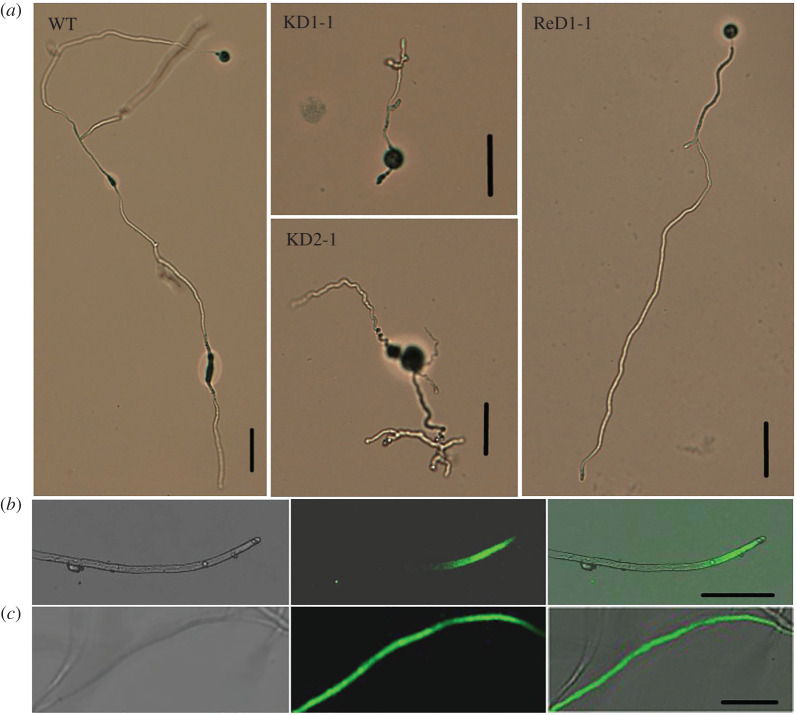
Microexamination of mycelium development of the wild-type strain and *ΔPcDHCR7* transformants and green fluorescence localization in eGFP labelled transformants. (*a*) Morphology of different strains after 1 day's germination of cystospores. WT indicates the wild-type strain BYA5; KD1-1 and KD2-1 are representative *ΔPcDHCR7* transformants; ReD1-1 is a *PcDHCR7*-complemented transformant. (*b*,*c*) Typical green fluorescence localization of a representative transformant expressing PcDHCR7-eGFP (*b*) or eGFP protein (*c*). Bright field (left), GFP field (middle) and merged field (right) are shown. Bar, 50 µm.

The finding that the encysted zoospores from the knock-out strains showed normal germination rates but nevertheless failed to cause lesions led to the speculation that the development from germ tube into mature mycelium of knock-out strains was hampered. To confirm this, we first examined the development of the germ tube under the microscope. At 24 h after zoospore encystment and germination, the wild-type strain showed elongated hyphae with occasional branching (figure 6*a*). By contrast, in the knock-out strains, most germ tubes had stopped growing, and many hyphae were twisted with abnormal shaped tips and deformed branches (figure 6*a*). Complementation of the knock-out strain KD1-1 with *PcDHCR7* largely restored the wild-type morphology of the hyphae. This aberrant growth phenotype probably explains why zoospores of knock-out strains lost pathogenicity (figure 5*a*,*b*,*d*) and why the colonies of the knock-out strains have a smaller diameter when compared to the wild-type strain (table 1). Yet, the fact that the mycelium grows, albeit slower, and that there is sporulation, suggests that the growth retardation for the *ΔPcDHCR7* transformants mainly happens during the development from germ tube to mature mycelium. Once this developmental phase is passed, the knock-out strains can regain their strength and show a close to normal growth pattern. To test how this affects pathogenicity, we inoculated pepper leaves with mycelial plugs instead of zoospores. This head start indeed empowered the knock-out strains to cause lesions and even at a similar speed as the wild-type strain (figure 5*c*). This demonstrates that PcDHCR7 is crucial for germ tube development and growth during an early step in the infection process, probably after invasion at the inoculation site. To test whether this developmental defect in the *ΔPcDHCR7* transformants is due to the lack of sterols that results from PcDHCR7 activity in mycelium, brassicasterol was applied to zoospores prior to inoculation or germination. The results showed that the supplementary exogenous sterol could rescue neither zoospore pathogenicity nor mycelium development, suggesting that the developmental defect is not due to the lack of suitable sterols (electronic supplementary material, figure S5).

To better understand the role of PcDHCR7 in mycelium growth, a protein of PcDHCR7 with an eGFP tag [30] at the C-terminal was overexpressed in *P. capsici*, and transformants expressing the free eGFP protein were used as negative controls. The resulting green fluorescence signal mainly accumulated in apical and subapical parts of young mycelia in PcDHCR7-eGFP-expressing strains (figure 6*b*), whereas in eGFP-expressing strains, the signal was extensively distributed all over the mycelia (figure 6*c*). Taken together, PcDHCR7 is mainly localized in the metabolically active zone of the young mycelium and plays a key role in *P. capsici* mycelium development.

### 2.7. *Phytophthora capsici* may remodel its transcriptome to compensate for PcDHCR7 deficiency

**Figure 7.**
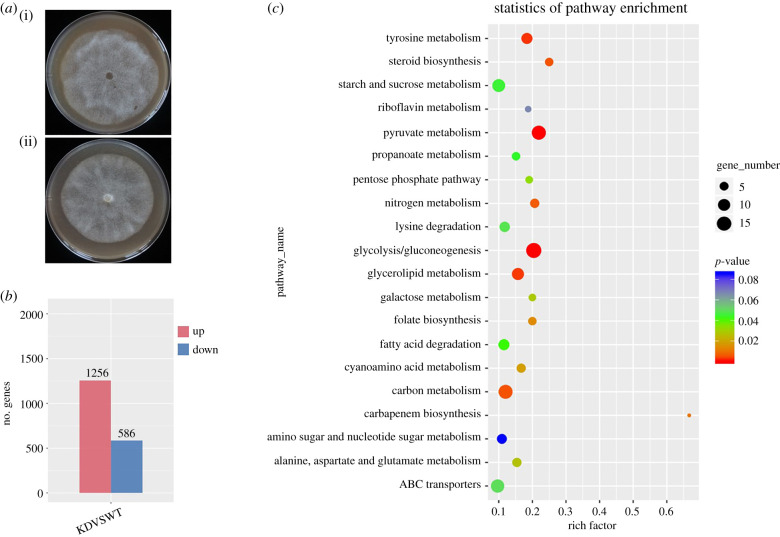
Comparison of the transcriptome in mycelium of the wild-type strain and a *ΔPcDHCR7* transformant. (*a*) Morphology of colonies of the wild-type strain BYA5 (i) and a *ΔPcDHCR7* transformant KD1-1 (ii) after 4 days of cultivation on V8 medium. (*b*) Number of genes that show at least a twofold increase (up) or decrease (down) in expression in the transformant KD1-1 when compared to the wild-type strain BYA5. (*c*) KEGG enrichment scatterplot showing the most significantly (top 20) changed KEGG pathways.

The *ΔPcDHCR7* transformants showed serious defects in the developmental shift from germ tube to the mature mycelium. However, apart from a slight growth reduction, colonies of the transformants did not display substantial differences from that of the wild-type strain, even though *PcDHCR7* displays the highest expression in mycelium (figure 1*c* and figure 7*a*). To further explore the effects of knocking out *PcDHCR7* at the molecular and cellular level, transcriptome profiles of mature mycelia of the wild-type strain BYA5 and a *ΔPcDHCR7* transformant KD1-1 were compared. Among the approximately 17 000 genes that were expressed in mycelium, 1256 genes were found to be upregulated in expression in the knock-out transformant when compared to the wild-type strain and 586 were down-regulated (figure 7*b*; Dataset 1). Two biological replicates showed a similar trend in the change of differentially regulated genes. Moreover, the transcriptome data were validated by qPCR of 12 randomly picked genes, including four upregulated and four downregulated genes, and four genes expressed at similar levels in wild-type and knock-out strains. This showed consistency in transcriptome and qPCR results for 11 out of 12 genes (electronic supplementary material, figure S6), indicating that the transcriptome data are reliable.

KEGG enrichment showed that the differentially expressed genes (DEGs) are involved in different pathways (figure 7*c*). Remarkably, many genes with a role in glycolysis/gluconeogenesis, tricarboxylic acid (TCA) cycle and pentose phosphate pathway were downregulated in the *ΔPcDHCR7* transformant KD1-1 (electronic supplementary material, figures S7, S8 and S9). This indicates that the transformant KD1-1 was growing in an energy-saving way, which was demonstrated by the fact that it produced less ATP and became more sensitive to respiration inhibitors (cyazofamid and fluazinam) when compared to the wild-type strain (electronic supplementary material, figures S10 and S11a,b). In addition, lots of amino acid and pyruvate metabolism-related genes were mostly downregulated (electronic supplementary material, table S1), probably due to a reduced activity in glycometabolism and the TCA cycle, the pathways that provide intermediates for the biosynthesis of amino acids such as pyruvate, acetyl-CoA and oxaloacetate [31]. Notably, many genes important for lipid metabolism, including those involved in glycerolipid metabolism, fatty acid degradation or biosynthesis, and ether lipid metabolism, have changed in their expression profile in the transformant (electronic supplementary material, table S2), suggesting a disorder in lipid homeostasis. Further investigation showed that the *ΔPcDHCR7* transformant was more resistant to a lipid synthesis inhibitor (propamocarb), when compared to the wild-type strain (electronic supplementary material, figure S11c). Apparently, the lack of PcDHCR7 affects lipid transport or utilization thereby causing lipid accumulation in mycelia. GO analysis showed that many of the differentially regulated genes participate in important cellular processes, such as metabolism, chromatin assembly or disassembly, transport, DNA integration, proteolysis, RNA-dependent DNA replication, electron transport, cell communication and protein amino acid phosphorylation (electronic supplementary material, figure S12). It seems that the absence of PcDHCR7 has an influence on several biological processes in the mycelium. Possibly *P. capsici* adopts a strategy to compensate for the defects caused by the loss of the PcDHCR7 by remodelling its transcriptome.

## 3. Discussion

Sterol synthesis involves a complicated pathway in eukaryotes, and a series of sterol synthesis inhibitors targeting different proteins in this pathway are widely used to combat pathogens [32,33]. Previous studies have shown that the sterol synthesis pathway has divergently evolved in different species within oomycetes, with only a few species sustaining such an ability [17,18]. Interestingly, some genes required for sterol synthesis including *DHCR7* are conservatively retained in the genomes of different sterol auxotrophic oomycetes, such as *Phytophthora* and *Pythium* species [9]. This raises the question why these oomycetes kept the sterol biosynthesis genes during evolution, since they are sterol auxotrophic and exploit exogenous sterols. To address this, we investigated the molecular and biological functions of DHCR7 in *P. capsici.* Our study showed that *PcDHCR7* is a bona fide gene that is expressed during growth *in vitro* and during pathogenesis *in planta*, with the highest expression in mycelium. We also found that *PcDHCR7* encodes a functional enzyme that has the predicted Δ7-sterol reductase activity, not only in a heterologous yeast expression system, but also in *P. capsici* itself. The enzyme activity enables *P. capsici* to convert inactive sterols to active ones for better use. Intriguingly, our study also revealed that PcDHCR7 seems indispensable in an important phase in the life cycle of *P. capsici.* In the absence of PcDHCR7, the transition from germ tube to mature mycelium is severely hampered. As a consequence, germ tubes emerging from encysted zoospores cannot establish a successful infection and the pathogenicity is lost. This suggests that PcDHCR7 also plays a key role in the development of *P. capsici*.

It has been shown previously that sterol auxotrophic *Phytophthora* spp. can exploit exogenous sterols for growth and reproduction [27]. The effect varies depending on the sterol structures and concentrations. For example, an early study found that 10 µg ml^−1^ of sitosterol showed the best promotion of vegetative growth and sexual production for *P. sojae* [28]. The current study demonstrated that the PcDHCR7 protein acts as the Δ7-sterol reductase in *P. capsici* itself and can transform inactive sterols into active ones. Others showed that *P. cactorum* can transform Δ5,7 sterols into Δ5 ones [34], and presumably this reduction is also mediated by DHCR7. Since *DHCR7* is conserved in all *Phytophthora* spp. analysed so far it is likely that the enzyme activity is also maintained in other species. The question that remains is to what extent *Phytophthora* spp. are exposed to sterols that need to be transformed by DHCR7 to be profitable for the pathogen. Plants possess a broad repertoire of sterols, with concentrations and ratios varying among species [35]. Moreover, the sterol composition of plants may change upon external stimulation, such as pathogen invasion [36]. Even though most plant sterols have already undergone the saturation mediated by DHCR7 and lost the double bond at the seventh carbon position, it is still plausible that *Phytophthora* spp. have retained *DHCR7* in order to deal with inactive sterols recruited from their hosts under certain circumstances.

By using CRISPR/Cas9 genome editing, we obtained *PcDHCR7* knock-out strains that showed normal mycelial growth albeit with a slightly reduced growth rate. Moreover, the knock-out strains showed normal asexual sporulation and cystospore germination, indicating that *PcDHCR7* is not essential for the survival of *P. capsici*. However, the knock-out strains showed serious defects in the development from germ tube to mature mycelium and, as a result, the knock-out strains were not capable to cause lesions on plants when zoospores were used for inoculating leaves. By contrast, infection was not hampered when mycelial plugs were inoculated and this is indicative of an indirect role for DHCR7 in pathogenicity. It is likely that the aberrant growth behaviour shortly after germination disables the pathogen to establish infection. Notably, one of the knock-out transformants, namely KD3-1, showed a more serious defect than the other two. This might be caused by one or more additional mutations in KD3-1, which could have been introduced by off-targeting, a negative side effect of major concern when using CRISPR-Cas for genome editing [37]. Since KD1-1 and KD2-1 have no such phenotypes, we assume that the additional defects in KD3-1 are not related to PcDHCR7 function. By *in situ* complementation, the defects in KD1-1 were largely restored despite the fact that the *in situ* complemented transformant that we obtained showed only half the expression level of that of the wild-type strain; this is in contrast with a recent study in which Qiu *et al.* [38] *in situ* complemented a *P. sojae* knock-out transformant, with the targeted gene encoding a regulatory B-subunit of protein phosphatase 2A and found the same level of expression as in wild-type *P. sojae*. This difference might result from changes in epigenetic markers on the chromatin, such as the N6-methyladenine (6 mA) modification in the DNA and histone methylation, which both play a key role in regulating gene expression in *Phytophthora* [39,40].

In the interplay between plants and pathogens, multiple factors may play a role in sterol homeostasis in both host and pathogen. One study by Gamir *et al*. [41] nicely demonstrated that a plant protein can hijack sterols in *Phytophthora brassicae.* The pathogenesis-related protein PR1, a well-known defence protein that is often upregulated upon pathogen attack, inhibits the development of *P. brassicae* in the stage from germ tube to colony. When treated with PR-1 germ tubes displayed an abnormal morphology and the sterol hijacking seems to happen inside the cell [41]. The abnormal morphology of the *ΔPcDHCR7* transformants might be caused by a similar change in sterol homeostasis as caused by PR-1 via hijacking sterols. Nevertheless, the developmental defects of *ΔPcDHCR7* transformants might also result from malfunctioning of other pathways or other compounds that are substrates of DHCR7. Jiang *et al*. [42] have used quantitative proteomics to compare embryonic brain tissue from a normal mouse and a DHCR7 mutant. They found many differentially expressed proteins with putative functions in multiple biological pathways, including mevalonate metabolism, apoptosis, glycolysis, oxidative stress, protein biosynthesis, intracellular trafficking and the cytoskeleton. Also in our transcriptome analyses, we identified many up- and downregulated genes in the *ΔPcDHCR7* transformant, indicating that various pathways are stimulated or inhibited because of the deficiency of the *PcDHCR7* gene. Mining these data and searching for correlations and patterns might provide clues for yet unknown functions of DHCR7*.* It is striking that the deficiency of PcDHCR7 only influenced a very specific but short phase in the development of *P. capsici.* The mature mycelium grew in a close to normal pattern but the stage after germination showed abnormal growth. The reason might be the difference in metabolism that produces the energy to support growth in that particular stage. In young mycelium and in the early infection stage, the main energy source is provided by fatty acid degradation, whereas in the mature mycelium, the energy production could be dependent on much more pathways [43]. The transcriptome data also indicated the notable change in lipid metabolism in the *ΔPcDHCR7* transformant. Although DHCR7 is known for its role in the biosynthesis of sterols, it may also interact with other lipids besides sterols. This could be a reason why lipid homeostasis was disrupted in the *ΔPcDHCR7* transformant thereby leading to a negative effect on glycometabolism and subsequently on the TCA cycle and amino acid metabolism [31]. Besides mining the omics data, more in depth analyses of the *ΔPcDHCR7* transformants at the cytological and biochemical level may reveal the role of this seemingly lonesome enzyme in sterol-auxotrophic *Phytophthora* spp. and this may answer the question why *Phytophthora* has retained the *DHCR7* gene.

## 4. Methods

### 4.1. Phylogenetic analysis of DHCR7 proteins

The protein sequences of DHCR7 homologues of representative evolutionary groups were acquired from different databases, including nine animals (*Homo sapiens*, *Pongo abelii*, *Bos taurus*, *Rattus norvegicus*, *Mus musculus*, *Cricetulus griseus*, *Xenopus laevis*, *Xenopus tropicalis* and *Danio rerio*), four land plants (*Arabidopsis thaliana*, *Ricinus communis*, *Glycine soja* and *Oryza sativa*), one diatom (*Thalassiosira pseudonana*), one brown alga (*Aureococcus anophagefferens*), five prokaryotes (*Legionella longbeachae*, *L. fallonii*, *Tatlockia micdadei*, *Parachlamydia acanthamoebae* and *Coxiella burnetii*), one virus (*Acanthamoeba polyphaga mimivirus*), and seven oomycetes (*P. capsici*, *P. sojae*, *P. ramorum*, *P. infestans*, *Pythium ultimum*, *Saprolegnia parasitica* and *Aphanomyces euteiches*). Most of the sequences were retrieved from the National Center for Biotechnology Information database (https://www.ncbi.nlm.nih.gov), except for *P. sojae*, *P. ramorum*, *P. infestans*, *Py. ultimum* and *Sa. parasitica* which were retrieved from the Ensembl protists database (https://protists.ensembl.org/index.html), for *P. capsici* which was retrieved from the JGI database (https://mycocosm.jgi.doe.gov/mycocosm/home), and for *A. euteiches* which was retrieved from the aphanoDB database (http://www.polebio.lrsv.ups-tlse.fr/aphanoDB/). The phylogenetic tree was constructed using Mega 6.0 [44]. The evolutionary history was inferred by using the maximum-likelihood method based on the JTT matrix-based model [45]. The online tool TMHMM Server v. 2.0 (http://www.cbs.dtu.dk/services/TMHMM/) was used for transmembrane domain analysis of PcDHCR7 protein.

### 4.2. RNA isolation and real-time-qPCR

To explore the expression profile of the *PcDHCR7* gene, biological materials from different developmental stages were collected, including zoospores, cystospores, germ tubes (about 5 hours after germination), 4-day-old mycelia from V8 medium, 4-day-old mycelia from minimal medium, mycelia with sporangia (4 days in the dark and another 5 days under light), and infection stage (4 days after inoculation on pepper leaves). Total RNA was extracted from the frozen samples using the SV Total RNA Isolation kit (Promega, Beijing, China), and cDNA was synthesized using the PrimeScript RT reagent Kit with gDNA Eraser (Takara, Beijing, China) according to recommended protocols. RT-qPCR was performed using a SYBR Premix Dimer Eraser kit (Takara, Beijing, China) on an ABI7500 sequence detection system (Applied Biosystems, United States). *Actin* and *WS21* genes were used as references for the normalization of the target gene expression [46]. The primers used for RT-qPCR are listed in the electronic supplementary material, table S3. The relative expression level was calculated with the 2^−ΔΔCT^ method [47], using the zoospore stage as a reference.

### 4.3. PCR and multiple sequence alignments

Regular polymerase chain reactions (PCR) were conducted using a 2 × master mix (Tsingke, Beijing, China), whereas those for plasmid construction were carried out using the high-fidelity DNA polymerase FastPfu system (TransGen, Beijing, China), following the recommended protocols. All of the primers used in this study are listed in the electronic supplementary material, table S3. Regular PCR was performed with the following programme: initial denaturing at 94°C for 4 min, followed by 34 cycles of denaturing at 94°C for 30 s, annealing at 55–65°C (depending on the primer) for 30 s and extension at 72°C for approximately 1 min for each 1 kb fragment, with a final extension at 72°C for 10 min. The resulting PCR products were sequenced by Tsingke (Beijing, China). Multiple sequence alignments of DNA sequences were carried out using the DNAMAN 9.0.1.116 software package (Lynnon Corporation), and alignment of amino acid sequences of DHCR7 protein from different oomycetes was performed with Clustal W [48].

### 4.4. *Saccharomyces cerevisiae* transformation

The plasmid pYES2/CT was firstly digested by *Eco*RI/*Xba*I (NEB) restriction enzymes. The coding DNA sequence (CDS) of *PcDHCR7* was amplified from cDNA of the *P. capsici* wild-type strain BYA5 and cloned into the plasmid using a modified In-Fusion HD Cloning method [49]. The *S. cerevisiae* strain BY4741 was used for heterologous expression of the gene *PcDHCR7*. The plasmid expressing *PcDHCR7* and the empty vector were, respectively, transformed into *S. cerevisiae* using the LiAc/SS carrier DNA/PEG method described in a previous study [50] with some modifications. Yeast cells were incubated overnight in 5 ml YPD medium at 30°C. The cultures were switched to 5–50 ml fresh YPD medium with an initial OD600 of 0.2 and were incubated at 30°C until the OD600 was approximately 1.0. The cells were collected from 5 ml cultures by centrifugation and washed with 0.1 M LiAc before they were resuspended in 26 µl sterile water, 240 µl 50% PEG (W/V), 36 µl 1.0 M LiAc, 50 ml ssDNA solution (2.0 mg ml^−1^, going through boiling in water and cooling on ice immediately before use) and 8 µl plasmid. The mixture was placed still on ice for 5 min and heat-shocked for 40 min at 42°C. Then, cells were collected by centrifugation, resuspended in 100 µl YPD medium and subsequently plated on SD agar plates without uracil. Transformation plates were placed at 30°C for 3–4 days, and colonies were confirmed by PCR and sequencing.

### 4.5. Sterol extraction and analysis

For sterol detection from *S. cerevisiae*, the yeast transformants were inoculated into a liquid SD medium and incubated for 2 days for inducing expression of the target gene. Then, yeast cells were collected and washed three times with sterile water. For sterol detection from *P. capsici*, the wild-type strain and a *ΔPcDHCR7* transformant were subcultured at least twice on minimal medium for *Phytophthora*; this medium does not include any sterols [51]. Then, they were transferred to minimal medium, which was modified with 20 µg ml^−1^ ergosterol and covered with one layer of cellophane. After four days of incubation at 25°C, the mycelia were collected. The yeast cells or *P. capsici* mycelia were dehydrated by freeze-drying, and 0.1 g dried sample was used for each detection. The cholesterol (50 µg) was added into yeast samples as an internal standard. The sterol extraction was performed using a protocol modified from a previous study [52]. After homogenization of the dried sample in 6 ml methanol-KOH (1%, w/v), saponification was conducted by heating in a water bath at 80°C for 90 min. The sample was cooled to room temperature, then 2 ml of double-distilled water and 4 ml n-hexane were added. The mixture was homogenized overnight in a shaker and then placed still for at least 30 min before the upper organic phase was transferred into a clean tube. The solvent was evaporated in a hydroextractor, and the residue was dissolved in 200 µl methylbenzene. The N,O-bis(trimethylsilyl)-trifluoroacetamide (BSTFA, 40 µl) was added to the sample, and the derivatization reaction was carried out for 60 min at 60°C in a water bath. For GC-MS analysis, a TSQ 8000 Evo gas chromatography in tandem with a triple quadrupole mass spectrometer was used. The sample (1 µl) was injected onto the column (Thermo TG-5MS, 0.25 µm, 0.25 mm × 30 m) with a helium flow rate of 1.2 ml min^−1^. The temperature program was as follows: 80°C for 1 min, followed by an increasing temperature of 12°C min^−1^ to 280°C (5 min) and another increasing temperature of 30°C min^−1^ to 290°C (5 min). The data were collected in SRM mode, and the following ion pairs (*m/z*) were used for the detection of different sterols: 458.5 > 368.4 and 368.4 > 353.4 for cholesterol; 470.5 > 380.4 and 470.5 > 365.4 for brassicasterol; 468.5 > 378.3 and 468.5 > 363.4 for ergosterol.

### 4.6. Genome editing and growth conditions of *Phytophthora capsici*

The wild-type *P. capsici* strain BYA5 was collected from an infected pepper sample in Gansu Province of China in 2011. The methods used for knocking out and complementation of the *PcDHCR7* gene have been described in a previous study [29]. The wild-type strain and transformants were all maintained on solid V8 medium at 25°C in the dark. For sterol treatment, the strains were subcultured on minimal medium without sterol at least twice before they were transferred to minimal medium modified with different concentrations of sterols.

Sporangia and zoospores of *P. capsici* were produced based on the protocol from a previous study [53]. Briefly, *P. capsici* strains were incubated at 25°C in the dark on solid V8 medium or minimal medium for 4 days, after which they were switched to a 12 L : 12 D h photoperiod for another 6 days. The sporangium production ability was evaluated by counting all the sporangia in the entire field of vision under a microscope with 100 times magnification. The plates were flooded with 10 ml sterile water and incubated at 4°C for 30 min and then at room temperature for 30 min. Zoospore production ability was determined by measuring zoospore concentrations using a hemocytometer. For cystospore germination evaluation, the zoospores were plated on 1% agar and dark-incubated at 25°C for 3–8 h until most of them from the wild-type strain had germinated. For mycelium development evaluation, the zoospores were plated on 1% water agar and after incubation for 1 day in the dark the hyphal growth was analysed by light microscopy. To evaluate the effect of exogenously added sterol on hyphal growth brassicasterol was applied to the zoospore suspensions to a final concentration of 20 µg ml^−1^, which were shaken vigorously and then kept still for 1 day before microscopy analysis. Each comparison was repeated at least three times. Single spore purification for complemented transformant was achieved by transferring individual germinated spores to fresh V8 plates, which were then dark-incubated at 25°C for 3 days.

For ATP content analysis, *P. capsici* was cultivated on solid V8 medium covered with a layer of cellophane in the dark for 4 days, after which the mycelia were collected and dried by removing the fluid. The ATP assay kit (Beyotime Biotechnology, Jiangsu, China), which is based on the luciferin-luciferase method [54], was used to quantify ATP in equal amounts of mycelia of the wild-type strain and the knock-out transformant. The relative ATP content was calculated in the transformant was by comparing the luminescent intensity of the transformant and the wild-type strain with four replicates.

For the sensitivity of *P. capsici* strains to different inhibitors, the wild-type strain and the knock-out transformants were grown on solid V8 medium supplemented with cyazofamid (10 µg ml^−1^), a respiration inhibitor that targets the Qi site of cytochrome bc1 complex [55] or with fluazinam (2.5 µg ml^−1^), another respiration inhibitor that is known to have uncoupling effect on oxidative phosphorylation respiration [56], or with propamocarb hydrochloride (50 µg ml^−1^), a widely used fungicide that affects lipid biosynthesis [57]. The compounds were dissolved in dimethyl sulfoxide (DMSO), and as control V8 medium without inhibitors containing a final concentration of 0.1% DMSO was used. The inhibition ratio was calculated by comparing the colony diameters on medium with and without inhibitors.

### 4.7. Infection assays

To compare the pathogenicity of the wild-type strain and that of transformants, zoospores and mycelia were used as inoculums, respectively. For zoospore inoculation, the concentration of different strains was adjusted to 20 000 zoospores/ml before 10 µl of zoospore suspension liquid was applied to the abaxial side of pepper or *N. benthamiana* leaves. To evaluate the effect of exogenously added sterol on pathogenicity brassicasterol was applied to the zoospore suspension to a final concentration of 20 µg ml^−1^. After 3 or 4 days, the infection lesions were measured. A recently developed method using red light imaging was used for infection evaluation and cell death visualization on *N. benthamiana* leaves [58]. On the other hand, 1 ml of zoospore suspension liquid was applied to the rhizosphere for each pepper seedling plant, which was 6–8 weeks old. After 5 days’ cultivation in a greenhouse, the morbidity and disease severity were investigated. For mycelium inoculation, the wild-type strain and transformants were incubated on solid V8 medium at 25°C in the dark, after which plugs (5 mm in diameter) with mycelia were cut from the edge of colonies and inoculated on the adaxial side of pepper leaves. After 4 days, the disease lesions were determined.

### 4.8. Statistical analysis

The data collected in this study were subjected to analysis of variance using DPS software v. 7.05. Differences between means were determined using Duncan's multiple range test at *p* = 0.01.

### 4.9. RNA-Seq and transcriptome analysis

For RNA sequencing, mycelium samples were collected from the V8 medium after 4 days of culturation. Total RNA was isolated from the samples using Trizol reagent (Invitrogen) and an RNA Clean-Up Kit-5 (Zymo Research, R1016), following the manufacturer's instructions. The extracted mRNA was used to construct the cDNA library, and the library construction was sequenced on an Illumina Hiseq 4000 Platform (Lc-Bio Technologies, Hangzhou, China). The preprocessed RNA-Seq reads were mapped to the reference genome of *P. capsici* strain LT1534 (https://mycocosm.jgi.doe.gov/Phyca11/Phyca11.home.html) [59] using the HISAT package [60], and the mapped reads of each sample were assembled using the StringTie method [61]. The DEGs were identified from RNA-Seq data with the cut-off of the corrected *p*-value < 0.05, using log2foldchange greater than or equal to 1 as a threshold. Analyses of the biological information of DEGs were performed using an online database (http://geneontology.org/); analysis of the KEGG (Kyoto Encyclopedia of Genes and Genomes) was performed using an online database (www.genome.jp/kegg) as a reference.

## Data Availability

The RNA-seq data have been deposited in NCBI Sequence Read Archive database, and the accession number is PRJNA788200. The data are provided in the electronic supplementary material [62].
